# Identification of the Molecular Basis of Nanocurcumin-Induced Telocyte Preservation within the Colon of Ulcerative Colitis Rat Model

**DOI:** 10.1155/2021/7534601

**Published:** 2021-07-29

**Authors:** Eetmad A. Arafat, Rehab Elsayed Marzouk, Sally Abdallah Mostafa, Walaa H. E. Hamed

**Affiliations:** ^1^Histology and Cell Biology Department, Faculty of Medicine, Mansoura University, Mansoura 35511, Egypt; ^2^Medical Biochemistry and Molecular Biology Department, Faculty of Medicine, Mansoura University, Mansoura 35511, Egypt

## Abstract

**Background:**

Telocytes (TCs) are a distinct type of interstitial cells that play a vital role in the pathogenesis of ulcerative colitis and colonic tissue hemostasis. The aim of this study was to examine the effect of nanocurcumin (NC) on the morphometric and immunohistochemical characterization of TCs in the ulcerative colitis (UC) rat model.

**Methods:**

Forty rats were randomly divided into control, NC, UC, and UC+NC groups. At the end of the experiment, the colon was dissected and prepared for histopathological and immunohistochemical assessment. Tissue homogenates were prepared for real-time PCR assessment of interleukin-6 (IL-6), tumor necrosis factor-alpha (TNF-*α*), and transforming growth factor-beta (TGF-*β*) gene expression. Our results revealed extensive mucosal damage with inflammatory cell infiltration, significant reduction of CD34, and vimentin immunostained TCs in the colon of the UC group with significant elevation of expression of IL-6, TNF-*α*, and TGF-*β*. The UC+NC-treated group revealed significant elevation of TC count compared to the UC group besides, a significant reduction of the three gene expression.

**Conclusion:**

NC successfully targeted the colonic tissue, improved the mucosal lesion, preserve TCs distribution, and count through its anti-inflammatory and fibrinolytic properties.

## 1. Introduction

Ulcerative colitis (UC) is a subtype of inflammatory bowel disease (IBD), frequently affecting the rectum and left colon [1]. Although it has been shown that hereditary, immunological, and environmental elements are involved in the pathogenesis of UC, the specific etiology of the disease remains unknown [2]. Certain key roles involved within the signaling pathway in the development of IBD are performed via molecular mechanisms that are not yet fully understood [3].

There is growing evidence that cytokine production plays an important role in IBD. The pathological characteristics of the disease include increased inflammatory cell infiltration and proinflammatory mediators such as tumor necrosis factor-alpha (TNF-*α*) and interleukins, in addition to an excessive release of reactive oxygen species and reactive nitrogen species with loss of antioxidant abilities of the colon and mucosal continuity [4].

One of the first-line treatments for UC is anti-inflammatory drugs, including corticosteroids and aminosalicylates. Despite their high effectiveness, these drugs have extensive side effects [5]. Thus, a more reliable and less risky anti-inflammatory treatment is needed.

Disturbed colonic motility is well established in patients with UC, and dysmotility has been associated with changes in the enteric neuromuscular compartment secondary to the fibrotic colon as well as the destruction of interstitial cells [6].

Telocytes (TCs) are a distinct subset of interstitial cells characterized by small cell bodies and relatively long and thin extensions called telopodes (Tps) [5, 7–9]. TCs were shown to occupy a strategic position in relation to stem cell niches and precursor stem cells [8]. In the colon, they form a subepithelial reticular network that is present under the intestinal crypts, and they are involved in supporting the stem cell niche of this region [10]. TCs are also thought to play a major role in tissue repair/regeneration, in addition to forming intercellular networks within the muscularis externa, which consequently supports peristaltic movements [11].

TCs and their Tps were identified in close contact with other TCs via direct cell-to-cell connections, stem cells, inflammatory cells, nerves, the microvasculature, immune cells, and noncellular elements, such as collagen and elastic fibers [11].

The lamina propria of the gut contains TCs exhibiting differential upregulation of IL-6 and IL-10, which are thought to be involved in many autoimmune and inflammatory diseases [12].

Curcumin is a natural spice and food coloring herb that is frequently used in traditional medicine. Several previous studies have shown that curcumin extract from turmeric rhizomes has anti-inflammatory, antioxidant, anticancer, antimicrobial, and wound healing properties [13]. However, the primary restriction of its medicinal use is that curcumin is extremely hydrophobic with poor water solubility. Therefore, it exhibits poor absorption from the gastrointestinal tract and low bioavailability following oral intake [14]. Several studies have been conducted to improve the oral bioavailability of curcumin. Nanotechnology was indicated as one of the most effective methods to enhance the bioavailability of curcumin after oral administration, which was 30-fold higher than that of powder form in both rats and humans [15].

Although the anti-inflammatory and fibrinolytic properties of nanocurcumin are well established, there is no sufficient molecular or cellular basis for its impact on the organization and morphometric or immunohistochemical characterization of TCs. To the best of our knowledge, this is the first basic research conducted upon nanoparticle curcumin investigating the morphometric analysis of TCs in an UC model.

## 2. Materials and Methods

### 2.1. Preparation of Curcumin Nanoparticles

The hydrothermal squeezing method was implemented for the preparation of curcumin nanoparticle extract. A total of 10 g of nature-made turmeric curcumin powder (Sigma-Aldrich, St. Louis, MO, USA) was dissolved in a 100 ml saline solution containing 2% Tween 80 in a 200 ml Teflon beaker. It was then autoclaved at 40°C overnight for approximately 24 h. Curcumin nanoparticles were squeezed from the powder and suspended within the solution under the effect of temperature and pressure in the closed autoclave. The obtained suspension was then diluted to a precalculated concentration for administration. The stock solution was stored in a cold dark place until use [16].

Nanoparticles were characterized using a transmission electron microscope and Fourier-transform infrared (FTIR) spectroscopy in the wavenumber range of 400–4000 cm−1 and accuracy of 1.0 cm−1 (Tensor 27, Brucker, Germany).

### 2.2. Experimental Animals

Forty adult male Wistar rats (weight, 120–150 g; 8–10 weeks old) were obtained from the Animal House, Faculty of Pharmacy, Mansoura University. Rats were housed in sanitary well-ventilated steel cages (two rats per cage). They were left to acclimatize for 1 week before beginning the experiment. Animals were fed ad libitum under standard laboratory conditions with 12 h light/dark cycles and a temperature-controlled environment (20 ± 2°C).

### 2.3. Experimental Induction of the UC Model

One day before the induction of colitis, rats were starved but allowed free access to drinking water. After light ether anesthesia, acute colitis was performed through intrarectal administration of 1 ml of 4% acetic acid solution (AA) (Sigma-Aldrich) via a soft silicone catheter, and then 2 ml of air was injected in the catheter to allow the colonic spread of AA. Lastly, rats were held straight for 2 min to avoid any leakage from the anus [17, 18].

### 2.4. Experimental Design

A larger sample size than that estimated from the “resource equation” method was used to overcome predictable animal deaths from UC or the experimental techniques.

Rats were divided into four groups (10 rats each). The control group (negative control) each received a single intrarectal administration of 2 ml saline. NC-treated group (the positive control) each received 100 mg/kg nanocurcumin orally through a gastric tube daily for 2 weeks.

The ulcerative colitis group (UC group) included ten rats that underwent induction of UC via intrarectal injection of 2 ml 4% AA as described above. UC+NC group included ten rats that underwent induction of UC and received daily oral gavage of 100 mg/kg nanocurcumin for 2 weeks [19].

### 2.5. Tissue Sampling and Staining

Following 24 h from the end of experiment, all rats from the four groups were euthanized via intraperitoneal injection of sodium pentobarbital anesthesia (50 mg/kg). The abdomen was dissected, and the left colon was divided into three segments, followed by fixation of one segment from each animal in 10% buffered formalin overnight for preparation of paraffin blocks. A rotatory microtome was used to obtain 5 *μ*m-thick sections to stain as follows: hematoxylin and eosin (H&E) for histological evaluation, Masson's trichrome staining for evaluation of fibrous tissue, and immunohistochemical staining with CD34 and vimentin for identification of TCs.

Another colon segment was fixed in glutaraldehyde and osmic acid for capsule preparation, and semithin sections stained with toluidine blue were obtained.

The final segments were immediately submerged in RNA later (Qiagen, Germany), incubated at 4°C overnight or longer, followed by total RNA extraction the next day.

### 2.6. Immunohistochemical Study

Paraffin sections (5 *μ*m thick) mounted on positively charged glass slides were deparaffinized and hydrated. Endogenous peroxidase activity was blocked by immersing the slides in 10% H2O2. The antigenic sites were unmasked by submerging the sections in 0.001 mol (M) citrate buffer (pH = 6) followed by boiling in a microwave for 5 min. Nonspecific staining of the slide background was avoided by incubating the slides with 1/100 normal rabbit serum for 20 min. Slides were then washed in 0.1 M phosphate-buffered saline (PBS) for 5 min. Diluted primary antibodies, including anti-CD34 (polyclonal, 1 : 100, Product# PA5-78978, Thermo Fisher Scientific) and rat polyclonal Vimentin (1 : 100; Cat #PA5-27231, Thermo Fisher Scientific), were then added to the slides for 1 h followed by washing with PBS. a secondary antibody (1 : 2000, Vector Laboratories) was added to the slides for 10 min. Avidin–biotin–peroxidase complex (Vector, Burlingame, CA) was applied for 30 min to visualize the site of immunostaining reaction, while 3,3-diaminobenzidine was administered for 10 min as a chromogen. Lastly, Mayer's Hematoxylin was used as a counterstain. Negative control sections were generated as outlined above with replacement of the 1ry antibodies with phosphate-buffered saline. The capillary endothelium and tonsil tissues were sectioned and stained as positive controls for CD34 and Vimentin, respectively.

### 2.7. Morphometric Study

Quantitative assessment of CD34- and vimentin-positive TCs was performed on colonic sections immunologically stained with anti-CD34 and antivimentin antibodies using a CX31 light microscope (Olympus, Japan) connected with Leica Qwin 500 image analyzer computer system (Leica, England). Serial sections were obtained from the colon of each rat (6 rats|group), and 5 slides were selected from each rat (one|ten serial sections). Sections were examined at 400X. The examined fields included the mucosa, submucosa, circular muscle layer, myenteric plexus, and longitudinal muscle layer. Counting was conducted manually, including only all the cells with definite nuclei. The area percentage of collagen fibers was measured using five slides for each rat at 250X.

### 2.8. Biochemical Study

Determination of IL-6, TNF-*α*, and TGF-B genes expression by real-time PCR (RT-qPCR).

RNA purification was performed using TRIzol reagent (Invitrogen, USA). Complementary DNA was then synthesized using Thermo Scientific Maxima First Strand cDNA Synthesis Kit with dsDNase (Thermo Fisher Scientific, Rockford, USA).

Primer sets for genes were designed using the Primer3PLUS software (v. 0.4.0; http://frodo.wi.mit.edu/;Table 1). Real-time PCR assays were performed using the Applied Biosystem 7500 real-time PCR detection system (Life Technologies, USA) according to the method described [20] with the SensiFAST SYBR Lo-ROX PCR Master Mix Kit (Bioline, United Kingdom). The total reaction volume was 20 *μ*l, and the thermal reaction profile was as follows: initial denaturation at 95°C for 2 min and then 40 cycles of 95°C for 5 s followed by 60°C for 30 s. Calculation of fold change of gene expression was normalized to *β*–actin according to cycle threshold (Ct) method (2^-*ΔΔ*Ct^). The relative gene expression for all genes was normalized to one in the control group (negative control) [21].

### 2.9. Statistical Analysis

Morphometric and molecular results were statistically analyzed using the Statistical Package for the Social Sciences software (version 21). Data are presented as mean ± standard deviation, and *P* values < 0.05 represent significant differences.

## 3. Results

### 3.1. Characterization of Curcumin Nanoparticles

**Figure 1:**
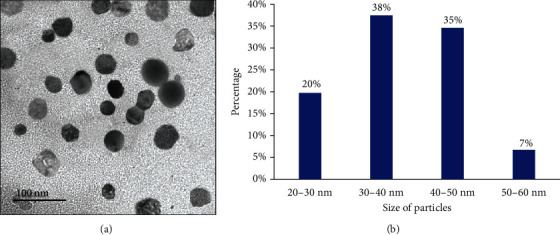
(a) Photomicrographs of the transmission electron microscope of nanocurcumin particles showing their average diameters. (b) Histogram represents the relation between nanocurcumin size and the count %.

**Figure 2:**
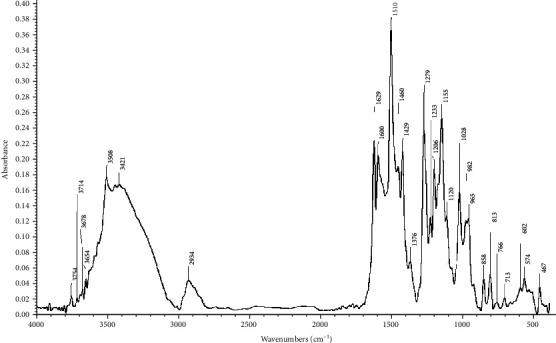
Fourier-transform infrared (FTIR) spectrum of the nanocurcumin.

Transmission electron microscopic study of curcumin nanoparticles used in this study revealed an average nanoscale diameter less than 50 nm (Figure 1). FTIR spectrum analysis revealed numerous absorption bands indicative of the presence of curcumin (Figure 2).

### 3.2. Histopathologic and Morphometric Results

#### 3.2.1. (H&E) Staining Results

**Figure 3:**
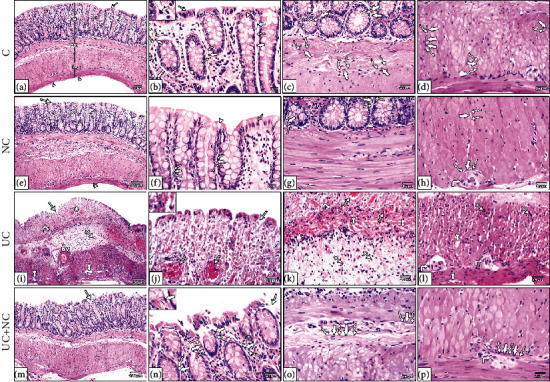
Photomicrographs of H&E-stained sections in the colon. (a–d) The control group and (e-h) NC group both show the normal structure of the colon; the mucosa (M), submucosa (SM), musculosa (Ms), and serosa (double arrowheads). The mucosa has regularly arranged closely backed tubular crypts (C) the crypts lines by simple columnar absorptive cells (arrow) with numerous goblet cells (arrowhead). The surface shows intact surface epithelium, and the glands are resting on a thin layer of muscularis mucosa. A thin layer of lamina propria containing numerous lymphocytes is present between crypts. (i–l) The ulcerative colitis group reveals loss of normal mucosal architecture, disturbed crypts (star), loss of surface epithelium and goblet cells (arrow), dilated congested blood vessels (lined arrow), and areas of mucosal ulceration (arrow) with thickening of the underling muscularis mucosa (curved arrows). The submucosa is wide with thick dilated blood vessels (BV). Inflammatory cell infiltration (IFC) is obvious in the mucosa and submucosa. The musculosa reveals separation between muscle bundle (tailed arrow), inflammatory cell infiltration (crossed arrow), and myenteric plexus (right-angled arrow). (m–p) The UC+NC group shows the normal architecture of the colon apart from narrow areas of interrupted surface epithelium (arrow). Note: telocytes appear as spindle shape cells (thick arrow) with small flat nuclei and long telopodes (wavy arrow). (H&E, (a, e, i, and m) X100, (b, c, d, f, g, h, j, k, l, n, o, and p) X400 insert in b, j, and n X1000).

H&E-stained sections of the control group colon sections exhibited the same histological picture in both the control and NC groups. Colons consisted of a normal histological arrangement of the four layers of the tissue wall: mucosa, submucosa, muscularis externa, and serosa. The mucosa consisted of regularly arranged, closely backed intestinal crypts lined by columnar absorptive cells with abundant goblet cells. A thin layer of lamina propria with few lymphocytes was observed, as well as TCs in sections presenting as spindle shape cells with long Tps. These cells were noted to form a network around the crypts in the lamina propria, in the submucosa, and in between the muscle fibers. The control group (Figures 3(a)–3(d)) NC group (Figures 3(e)–3(h)).

Histological examination of H&E-stained sections from the UC group revealed extensive mucosal damage in the form of loss of columnar absorptive epithelium lining the surface, few crypts with few goblet cells, heavy infiltration with inflammatory cells, and numerous congested blood vessels with areas of hemorrhagic lesions. Other areas with complete loss of mucosal glands and hypertrophy of the underlying muscularis mucosa were observed. The submucosa revealed diffuse mononuclear cellular infiltration, submucosal edema, and an obvious increase in submucosal thickness with dilated congested thick wall blood vessels. The muscularis externa exhibited separated muscle fibers with the presence of mononuclear cell infiltration. TCs were ill-distinguished in the sections (Figures 3(i)–3(l)).

The UC+NC group revealed a similar structure to that of the control group with apparent preservation of intestinal crypts, goblet cells apart from dispersed inflammatory cell infiltrations in the lamina propria. However, small areas with discontinuous surface epithelium were still observed. TCs with long Tps can be seen in the sections (Figures 3(m)–3(p)).

#### 3.2.2. Masson's Trichrome Staining Results

**Figure 4:**
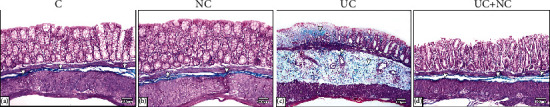
Photomicrographs of Masson trichrome stained sections in the colon. (a) The control group and (b) NC group both reveal fine collagen fibers (arrowhead) in the submucosa. (c) UC group shows a large amount of collagen fibers in the mucosa and submucosa. The collagen fibers in the submucosa are interrupted by edema. (d) UC+NC group shows fine collagen fibers in the submucosa (Masson trichrome X100).

**Figure 5:**
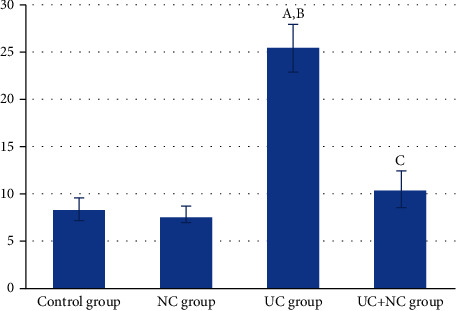
Analysis of the area percentage of collagen fibers in the study groups (6 rats/group). A: significant difference in relation to control group. B: significant difference in relation to NC group. C: significant difference in relation to UC group.

The control and NC groups revealed regularly arranged fine collagen fibers in the submucosal layer and in between the crypts of the mucosa (Figures 4(a) and 4(b)). The UC group revealed a noticeable deposition of large amounts of collagen fibers in the mucosa. The submucosa exhibited collagen fibers separated by edema and congested blood vessels (Figure 4(c)). The UC+NC group had mild collagen fibers at the same sites (Figure 4(d)). A statistically significant increase was observed in the percent area of collagen fiber in the UC group compared with the control group with a significant decrease in the UC+NC group compared with the UC group (Figure 5).

### 3.3. Immunohistochemical Results

**Figure 6:**
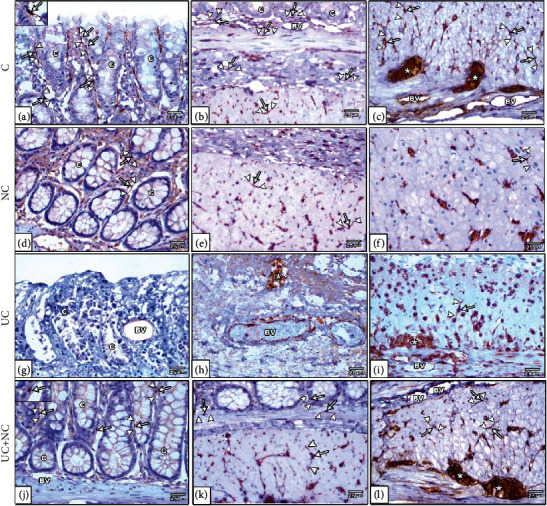
Photomicrographs of CD34 immunohistochemical stained sections in the colon. (a–c) The control group and (d-f) NC group both show numerous CD34 positive TCs (arrow) with long Tps (arrowhead) in the lamina propria close to the crypt epithelium, in between smooth muscle of muscularis mucosa, submucosa, between SMF of musculosa, in the myenteric plexus (star) and around blood vessels (BV). (g–i) The UC group reveals undetected TCs in the mucosa and submucosa. Few numbers of TCs are present in the musculosa and myenteric plexus (star). (j–l) The UC+NC group shows numerous TCs in all layers of the colon. Note: the endothelium of blood vessels (BV) reveals CD34 positive immune stain (CD34 immunostaining X400 insert in a and j X1000).

**Figure 7:**
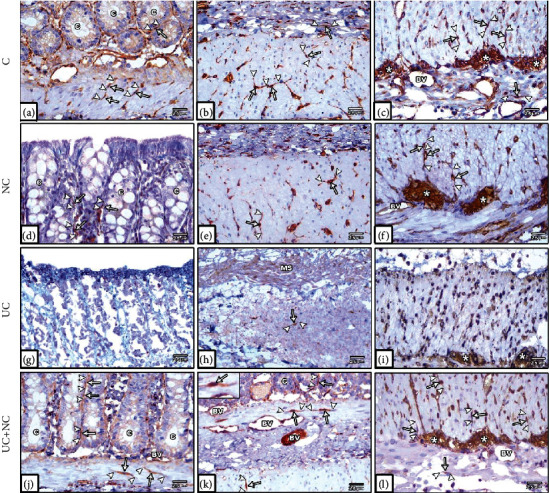
Photomicrographs of vimentin immunohistochemical-stained sections in the colon. (a–c) The control group and (d–f) NC group both show plentiful vimentin-positive TC cells (arrow) with long Tps (arrowhead) close to the crypt epithelium, muscularis mucosa, submucosa, musculosa, myenteric plexus, and around blood vessel (BV). (g–i) The UC group reveals hardly seen TCs in the mucosa and submucosa and few numbers of cells in the musculosa and myenteric plexus. (j–l) The UC+NC group shows abundant TCs in the mucosa, submucosa, musculosa, and myenteric plexus (∗) (vimentin immunostaining X400 insert in k X1000).

Immunostained sections from the colon of the control and NC group revealed numerous CD34+ and vimentin-positive TCs. The cells were cylindrical with thin, long Tps. In the mucosa, some TCs were observed in the lamina propria close to the crypt epithelium. TCs were also distributed among the smooth muscle cells of the muscularis mucosae. In the submucosa, TCs were distributed among collagen and elastic fibers in close contact with the blood vessels. In the muscularis propria, TCs were distributed among the smooth muscle bundles of the circular and longitudinal layers and surrounding the myenteric plexus. They were also found in the connective tissue and around the blood vessels of the subserosal layer (Figures 6(a)–6(f) and 7(a)–7(f)).

**Figure 8:**
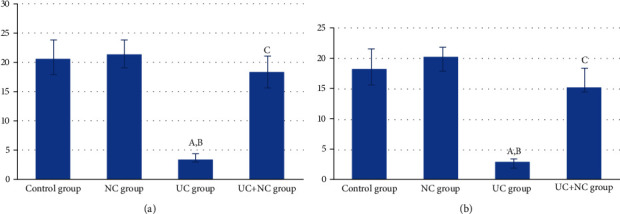
(a) Analysis of the number of CD 34 positive immune-stained TC/HPF in the study groups (6 rats/group). A: significant difference in relation to control group. B: significant difference in relation to NC group. C: significant difference in relation to UC group. (b) Analysis of the vimentin-positive immune-stained cells TC/HPF in the study groups (6 rats/group). A: significant difference in relation to control group. B: significant difference in relation to NC group. C: significant difference in relation to UC group.

CD34- and vimentin-stained sections from the UC group revealed that TCs were rarely detected in the mucosa and submucosal layers. The muscularis externa revealed few cells (Figures 6(g)–6(i) and 7(g)–7(i)). A significant decrease in numbers of TCs was observed in the UC group compared to controls (Figures 8(a) and 8(b)).

The UC+NC-treated group revealed many CD34-positive TCs (Figures 6(j)–6(l)) and vimentin immune-stained cells (Figures 7(j)–7(l)) with similar distribution to that of the control group. A significant increase in the number of CD34 and vimentin immune-stained TCs was observed in comparison to the UC group, with no significant change when compared to controls (Figures 8(a) and 8(b)).

### 3.4. Semithin Section Results

**Figure 9:**
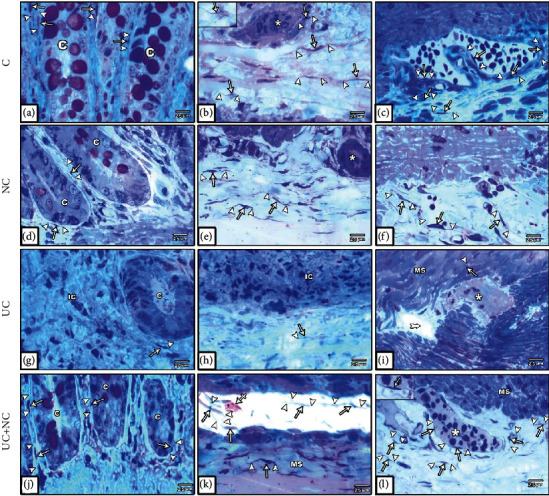
Photomicrographs of toluidine blue-stained semithin sections in the colon. (a–c) The control group and (d–f) NC group both show numerous TCs (arrow) with long Tps (arrowhead) around the intestinal crypts (C), in the submucosa, musculosa, and myenteric plexus (star). (g–i) The UC group reveals hardly seen TCs with short Tps round the remnant of crypts (C) and completely lost in inflamed areas (IC). Few TCs are hardly seen in the submucosa, the musculosa (MS), and myenteric plexus (star). (j–l) The UC+NC group shows numerous TCs with long Tps around the crypts (C) in the submucosa in contact with Mast cell (crossed arrow), the musculosa (MS), and myenteric plexus (star), (toluidine blue X400 insert in b and i X1000).

Histological examination of toluidine blue staining of semithin sections from both the control (Figures 9(a)–9(c)) and NC groups (Figures 9(d)–9(f)) revealed numerous small cylindrical TCs with long Tps forming a reticulum around crypts, in the submucosa, and in contact with smooth muscle fibers, blood vessels, and the myenteric plexus. The UC group exhibited few TCs around the remnants of intestinal crypts, and these were completely absent within inflamed and fibrotic areas. TCs were rarely seen in the submucosa, muscularis externa, and around the myenteric plexus (Figures 9(g)–9(i)). The UC+NC group revealed frequent TCs in the mucosa surrounding the crypts, in the submucosa with close contact to mast cells, and in close proximity to muscle fibers and the myenteric plexus (Figures 9(j)–9(l)).

### 3.5. Molecular Results

**Figure 10:**
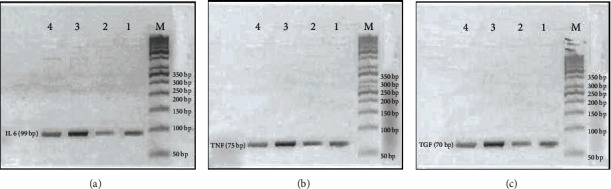
RT-PCR product of Il-6, TNF-*α*, and TGF-*β* genes. In all pictures, M lane for DNA ladder 50 bp, lane 1 control group (negative control), lane 2 NC group (positive control), lane 3 UC group, and lane 4 NC++ UC group. (a) is for IL 6 gene with product length 99 bp. (b) is for TNF-*α* with a product length of 75 bp. (c) is for TGF-*β* with product length 70 bp.

**Figure 11:**
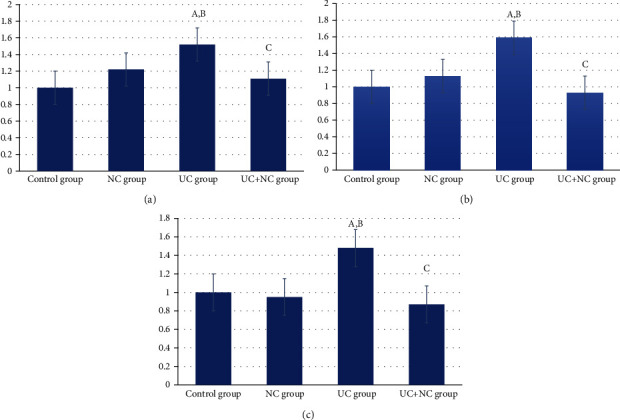
(a) Analysis of IL-6 within the control and the experimental groups. A: significant difference in relation to control group. B: significant difference in relation to NC group. C: significant difference in relation to UC group. (b) Analysis of TNF alpha within the control and the experimental groups. A: significant difference in relation to control group. B: significant difference in relation to NC group. C: significant difference in relation to UC group. (c) Analysis of TG-*β* within the control and the experimental groups. A: significant difference in relation to control group. B: significant difference in relation to NC group. C: significant difference in relation to UC group.

The levels of mRNA gene expression of TNF-*α*, IL-6, and TGF-*β* revealed statistically significant increased expression in the UC group (1.59 ± 0.26, 1.52 ± 0.34, and 1.48 ± 0.32, respectively) compared to that of the control group (1.13 ± 0.15, 1.22 ± 0.26, and 0.95 ± 0.20, respectively) with *P* values of 0.002, 0.022, and 0.005, respectively. No significant changes were observed between the UC+NC group and control groups. A statistically significant decrease in the expression of these three genes within the UC+NC group in comparison to the UC group was observed with *P* values of < 0.001, 0.011, and 0.004, respectively (Figures 10 and 11(a)–11(c)).

## 4. Discussion

The use of nanotechnology in the treatment of gastrointestinal diseases has recently been taken under serious consideration, since it is one of the most promising delivery systems for curcumin in terms of overcoming the issue of limited bioavailability.

Considering the potential effects of curcumin, in this study, we attempted to elucidate the anti-inflammatory and fibrinolytic properties of curcumin nanoparticles in a rat model of UC and correlated these properties with histomorphometric changes in TCs.

We hypothesized that the administration of AA allows for and benefits experimental induction of inflammatory and fibrotic models of UC. In support, previous studies [22, 23] have recently reported that intracecal administration of AA is associated with increased gene expression of caspase-1, IL-B1, and the NLRP3 inflammasome, giving a typical picture of UC.

In the present study, microscopic examination of the colon mucosa in the UC group revealed a disturbed architecture in the form of mucosal ulceration, goblet cell depletion, dilated congested blood vessels, loss of crypts, thick muscularis mucosa, and excess inflammatory cell infiltration. Analysis of the submucosa revealed significant edema and inflammatory cell infiltration. Our results were in line with previous work [24, 25].

RT-PCR analysis revealed significant elevation of TNF-*α* and IL-6. In the context of UC, both TNF-*α* and IL-6 are particularly important. IL-6 is a proinflammatory and anti-inflammatory cytokine. Excess production of IL-6 is involved in the pathogenesis of IBD with consequent accumulation of T cells in the intestinal wall. In addition, excess release of cytokines may result in disturbed tight junctions and, consequently, intestinal barrier damage [26, 27].

TNF-*α* is a proinflammatory cytokine associated with increased levels of IL-1, which stimulates leukocyte migration to the site of inflammation and increases the levels of metalloproteinases in the matrix that is responsible for the destruction of the intestinal extracellular matrix [28]. TNF-*α* stimulates the release of oxidative inflammatory mediators even further than proinflammatory cytokine [28].

Histopathological examination of UC+NC-treated rats revealed preserved cytoarchitecture of the colonic mucosa with an obvious decrease in inflammatory cell infiltration. These histological results can be explained by the significant reduction of IL-6 and TNF-*α* gene expression in the nanocurcumin-treated group. Our results are in line with those previously reported [29], and they documented that curcumin exhibited a potential anti-inflammatory effect and downregulated proinflammatory mediators (i.e., NO, PGE2, TNF-*α*, and IL-6) through the inhibition of NF-*κ*B and suppressed TLR4 activation.

Histological examination of Masson's trichrome-stained sections of the UC group revealed a significant increase in collagen deposition in the mucosa and submucosa of acutely inflamed colons of the UC group compared to the control groups. This histological picture was confirmed by RT-PCR analysis of TGF-*β*, which revealed a significant increase compared to the control group. TGF-*β* stimulates collagen formation via myofibroblast activation [30].

A significant reduction in collagen deposition and submucosal thickness was observed in the UC+NC-treated group, and these results were confirmed by a significant decrease of TGF-*β* level in these rats when compared to the UC group. This result is in line with the data of a previous research work [31], and they reported that curcumin decreased both TGF-*β* and collagen I expression and could consequently prevent or postpone myofibrogenesis. In addition, nanocurcumin prompted myofibroblast apoptosis [32] and can prevent fibrosis by decreasing the expression of cytokine and chemokine genes that are directly implicated in fibrosis [33].

Telocytes have been recognized in many vertebrates within several different organs. Until now, the identification of TCs remains under debate and an association between these cells and other interstitial cells is common. However, light microscopic recognition of TCs depends on their ability to form 3D stromal networks via their long telopodes [34]. TCs express different immunological markers according to the organs in which they reside and the function performed by these cells [11]. Recently, CD34 and vimentin have been considered as reliable markers for the identification of TCs in the gut. CD34 is a transmembrane phosphoglycoprotein generally recognized in hematopoietic stem cells and vascular endothelial cells [35]. Some researchers refer to TCs as CD34+ stromal cells [36, 37]. Vimentin is an intermediate filament that is widely expressed in endothelial cells within blood vessels, epithelial cells, some vascular smooth muscle cells, cartilage, and bone cells. It is also responsible for providing structural support to the tissue [38].

Regarding the distribution of TCs in the colon of the control group, they were found to form an extensive network around the crypts and in the lamina propria. This arrangement ensures the maintenance of tissue integrity under stress from mechanical forces, such as distention and contraction [34]. In the muscularis mucosa and the musculosa, both CD34 and vimentin revealed TCs as positive immune-stained cells with their processes intermingled between muscle bundles and all around the myenteric plexus. This could explain the role of TCs in the regulation of gastrointestinal motility together with the interstitial cells of Cajal [39]. The distribution of TCs in the rat colon is in line with that described in the gut of the grass carp and other tubular organs in mammals [40].

In the UC group, TCs were significantly decreased, especially in inflamed or fibrotic areas. This could be due to the change in the composition of the extracellular matrix (ECM) and excess collagen fiber deposition, with the fibrotic extracellular matrix entrapping the TCs and leading to cell death as previously documented in cases of heart failure [41], scleroderma, and IBD [42]. Recently, a research work reported that the proliferative ability of TCs decreased with increasing levels of TNF-*α* [43], which is in line with our results.

TCs were reported to play an important role in the gastrointestinal tract (GIT). They have direct contact with various types of immune cells, including mast cells, dendritic cells, lymphocytes plasma cells, eosinophils, basophils, and macrophages [9, 40]. In line with the proposed role of TCs in the regulation of immune response through the paracrine pathway, TCs in the uterus were postulated to play a role in peritoneal macrophage activation. The activated macrophages revealed more pseudopodia and secreted different inflammatory cytokines including TNF-*α*, IL1-R1, and IL-10, indicating an impending role of TCs in the mechanism of immunoregulation and immunosurveillance [44].

TCs have been proposed to play a role in tissue shaping through the regulation of ECM formation during organ development. Regardless of the significant reduction of telocytes in UC preceding fibrosis or being a consequence of tissue fibrotic, loss of TCs might be a factor in the altered organization of ECM in the intestinal wall [39]. Additionally, contact between fibroblasts and TCs has been reported in many organs [9], indicating a role of TCs in tissue homeostasis through the regulation of fibroblastic activity via inhibitory signals [39].

Our results showed that the UC group was associated with frequent watery diarrhea as compared with well-formed pellets in the UC+NC-treated group. This could be due to a reduction in TC numbers which has a strategic position between the smooth muscle bundles; besides, the inflammation observed in the mucosa and increased IL-6 and TNF-*α*. Of note, close contact between TCs and crypts stem cells and the secretion of Wnt protein from subepithelial telocytes is particularly important in stem cell differentiation and epithelial renewal [10], which is in line with our results indicating that loss of subepithelial TCs in the UC group led to the failure of ulcer healing and epithelial regeneration.

Based on the results of this study, TCs play an important role in the pathogenesis of UC. In agreement with this hypothesis, TCs in the lamina propria of the GIT have been reported to secreted high levels of IL-6 and IL-10 in comparison to the surrounding stromal cells [10], indicating a potential role of TCs in the pathogenesis of inflammatory and immune-regulatory diseases.

Limitation of the study, further ultrastructural and immunological studies are required for better understand the role of TCs in the pathophysiology of ulcerative colitis. In this work, we study the effect of short-term curcumin treatment in the UC rat model, and long-term treatment with nanocurcumin in chronic or relapsing UC models should be considered in future researches.

## 5. Conclusion

To the best of our knowledge, no available studies have evaluated the effect of nanocurcumin on colon telocytes within UC. In the present study, immunohistochemical examination of the nanocurcumin-treated group revealed a significant increase in the TC count compared with the UC group and no significant changes when compared with the control group. This could be due to the anti-inflammatory and antifibrotic effects of nanocurcumin. These immunohistochemical results were in parallel with the molecular assessment of IL-6, TNF-*α*, and TGF-*β*.

These results are indicative of a pertinent role of nanocurcumin as a remedy for UC through its beneficial anti-inflammatory and fibrinolytic properties. This study also shed light on the role of TCs in the pathogenesis of UC and colonic tissue hemostasis. This information may be beneficial to researchers in the future who may consider using TCs in tissue repair and regeneration as a possible alternative of cell therapies.

## Figures and Tables

**Table 1:** The sequences of the primer pairs of *β*-actin (as control gene), TNF-*α*, interleukin-6, and TGF *β* genes.

Gene	Forward primer	Reverse primer	Product length	Reference sequence
*β*–Actin	TACTGCTCTGGCTCCTAGCA	CGGACTCATCGTACTCCTGC	146 bp	NM_007393.5
TNF-*α*	GGCAGGTTCTGTCCCTTTCAC	TTCTGTGCTCATGGTGTCTTTTCT	75 bp	NM_001278601
Interleukin-6	TTCCATCCAGTTGCCTTCTTG	GGGAGTGGTATCCTCTGTGAAGTC	99 bp	NM_001314054
TGF-*β*	GAGGTCACCCGCGTGCTA	TGTGTGAGATGTCTTTGGTTTTCTC	70 bp	NM_0115772

## Data Availability

The data that support the findings of this study are available from the corresponding author upon reasonable request.
